# Enhancing cancer risk awareness and screening management through artificial intelligence: a narrative review

**DOI:** 10.3389/fonc.2025.1695749

**Published:** 2025-11-19

**Authors:** Jiaxuan Wu, Xiaolong Tang, Qian Zheng, Xinhang Gu, Li Ma, Jinghong Xian, Hui Mao, Jiadi Gan, Guiyi Ji

**Affiliations:** 1 Department of Pulmonary and Critical Care Medicine, West China Hospital, State Key Laboratory of Respiratory Health and Multimorbidity, Sichuan University, Chengdu, Sichuan, China; 2 Institute of Respiratory Health, Frontiers Science Center for Disease-related Molecular Network, West China Hospital, Sichuan University, Chengdu, Sichuan, China; 3 Precision Medicine Center, Precision Medicine Key Laboratory of Sichuan Province, West China Hospital, Sichuan University, Chengdu, Sichuan, China; 4 The Research Units of West China, Chinese Academy of Medical Sciences, West China Hospital, Chengdu, Sichuan, China; 5 Institute of Respiratory Health and Multimorbidity, West China Hospital, Sichuan University, Chengdu, Sichuan, China; 6 Department of Radiation Oncology, Shandong Cancer Hospital and Institute, Shandong First Medical University and Shandong Academy of Medical Sciences, Jinan, Shandong, China; 7 West China Outpatient Department, Sichuan University, Chengdu, China; 8 Health Management Center, General Practice Medical Center, West China Hospital, Sichuan University, Chengdu, China

**Keywords:** artificial intelligence, cancer, early screening, precise diagnosis, management

## Abstract

Cancer is a major public health problem worldwide, and early detection through risk awareness and screening is critical for improving patient outcomes. Although modern medicine has made certain progress, there are still many unmet clinical needs in areas such as precise diagnosis, precise treatment and risk assessment.Traditional strategies to promote public awareness and optimize screening programs face persistent challenges. With the development of modern science and technology, artificial intelligence (AI) has gradually become an important force driving innovation in the field of oncology.Recent advances in artificial intelligence, particularly large language models (LLMs), have introduced new opportunities to address these barriers by enabling personalized risk communication, predictive analytics, and automated decision support. By summarizing recent advances in the application of artificial intelligence to early cancer detection, this review seeks to propose innovative strategies for early screening and precise diagnosis, ultimately aiming to reshape the landscape of cancer prevention and treatment.

## 1 Introduction

Cancer remains one of the leading threats to human health worldwide, imposing a substantial burden on individuals and society (1, 2). Although medical advancements have enabled an increasing number of patients to receive effective treatment, their quality of life and long-term survival rate remain an important concern (3, 4).

Early detection refers to the early identification and intervention of cancers or precancerous lesions that can improve survival rates or reduce incidence rates, including screening for asymptomatic populations and diagnosis of cancer at an early stage (5). The purpose of early detection is to identify secondary cancers or precancerous lesions at the earliest point in time when intervention can improve survival rates or reduce incidence rates. Research and development in early cancer detection has brought substantial health benefits (6, 7); however, many cancers are still frequently diagnosed at advanced stages, where the prognosis is often poor. Patients with advanced-stage cancer may miss the optimal window for therapeutic intervention, and costly late-stage systemic treatments are commonly associated with severe side effects and poorer outcomes. Building on the success of early detection and expanding its application to other cancer types could significantly improve patient outcomes (8–10). Effective early detection strategies require careful consideration of the disease’s epidemiological characteristics, the accuracy and cost-effectiveness of detection technologies, patient acceptance, and the availability of medical resources. Under the premise of ensuring high diagnostic accuracy and a low rate of missed diagnoses, the selected detection technology should be as simple, cost-effective, and minimally invasive as possible (5).

Currently, cancer risk management faces three core challenges: insufficient public awareness (11, 12), screening accessibility differences (13, 14), and weak cross-sectoral collaboration (15). These challenges highlight that current cancer prevention and control efforts continue to face a substantial disease and economic burden—both of which are projected to increase in the future. The prolonged treatment duration and high costs associated with malignant tumors, along with the frequent hospitalizations of cancer patients, impose significant financial and psychological stress on patients and their families. Furthermore, these factors place a considerable strain on healthcare systems, the national economy, and overall social development. In light of this alarming situation, it is imperative to implement comprehensive cancer prevention and control strategies on a global scale.

With the continuous advancement of computer technology and statistics, doctors and computer professionals can now closely collaborate in areas such as early cancer screening and improving prognosis. Artificial intelligence encompasses technologies that employ computational systems to simulate human-like intelligent behaviors for problem-solving. In recent years, alongside advancements in computing power and the accumulation of massive datasets, the application of AI across diverse fields has advanced rapidly. Notably, within the medical domain, AI has demonstrated substantial potential and practical efficacy (16).

Currently, digital health, deep learning (DL) and LLMs are working together to expand the boundaries of cancer recognition and screening. For example, digital health platforms facilitate risk communication and real-time monitoring through mobile applications and wearable devices.AI-based imaging algorithms have demonstrated the ability to increase lesion-level cancer detection by 1.9- to 3.8-fold across multiple organ sites: prostate-MRI sensitivity rose from 67% to 88% with AI assistance (17), supplemental MRI triage driven by AI tripled the additional breast-cancer yield compared with standard density-only protocols (18), In addition, the latest LLMs have increased the accuracy of patient education, personalized Q&A, and real-time interventions to over 90%, while significantly reducing the communication time between doctors and patients (19–22).

**Figure 1:**
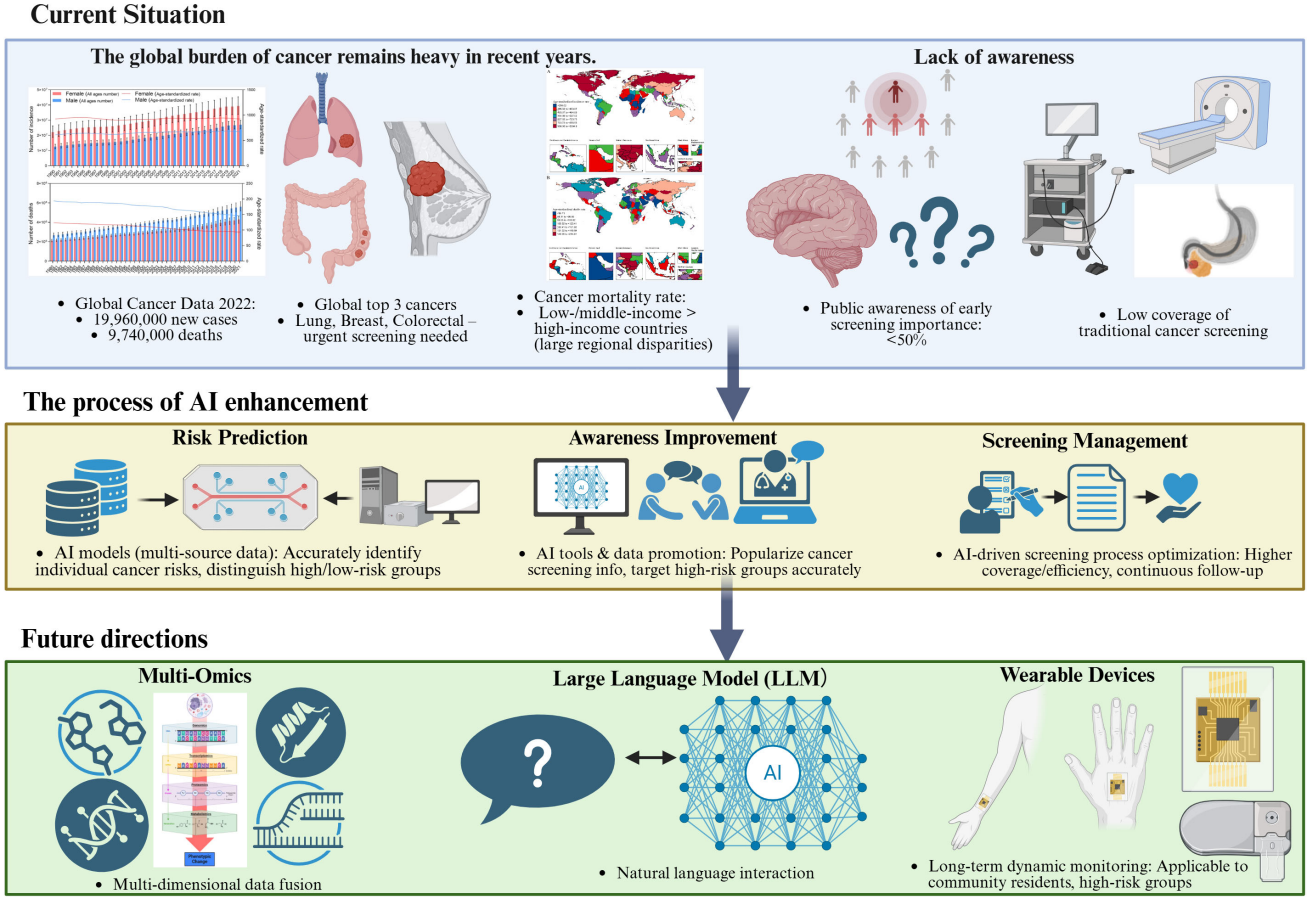
Conceptual framework of AI-enabled cancer awareness and screening: challenges, interventions, and future directions.schematic overview of the narrative review framework, illustrating three core components: (1) Top: Global cancer burden (incidence/mortality, high-priority cancers, regional disparities) and current gaps in awareness (low recognition of screening importance) and screening (low coverage, inefficient workflows, over/under-screening); (2) Middle: AI-enabled interventions targeting these challenges, including risk prediction (multi-source data integration), awareness enhancement (AI chatbots, targeted outreach), and screening management (AI-aided diagnosis, automated reminders); (3) Bottom: Future directions driven by multi-omics integration, large language models (LLMs), and wearable devices, which synergistically advance personalized and proactive cancer screening.

To address the key challenges outlined above, this review will focus on four main areas: (1) the current status of cancer risk factors and screening practices; (2) the application of artificial intelligence and digital technologies in cancer screening and management; (3) strategies to enhance public understanding of cancer risk factors; and (4) the future dierctions of AI in cancer risk awareness and screening management (**Figure 1**).

This study employed a narrative review approach guided by the PRISMA-Scoping Review framework to comprehensively explore the application of AI in enhancing cancer risk awareness and screening management. Relevant electronic databases, including PubMed, Scopus, and Web of Science, were systematically searched using appropriate keywords and controlled vocabulary terms related to “artificial intelligence,” “machine learning,” “deep learning,” “large language models,” “cancer risk,” “awareness,” and “screening.” Titles and abstracts were initially screened to identify potentially relevant studies, followed by full-text evaluation based on predefined inclusion and exclusion criteria. Studies were included if they focused on AI applications in cancer risk prediction, awareness improvement, or screening optimization, and were published in English. Studies unrelated to cancer prevention or screening, non-English articles, and those without full-text availability were excluded. The findings were synthesized and presented narratively, highlighting key themes, current challenges, and emerging trends in AI-driven approaches for improving cancer risk awareness and screening practice.

## 2 Cancer risk factors and screening: current landscape

The primary goal of cancer screening is to reduce mortality by detecting preinvasive or early-stage disease when treatment is most likely to be effective (23). In recent decades, significant progress has been made in cancer screening strategies. Across successive generations of clinical practice, screening guidelines for various cancer types have been continuously updated, enabling the earlier detection of lesions and thereby improving patient outcomes through timely intervention.

However, excessive or inappropriate screening may lead to unintended consequences, such as test-related complications, false-positive results, increased patient anxiety, and additional financial burden (23). Therefore, there is an urgent need to develop and adopt tailored screening strategies that are appropriate for the specific contexts of different countries and regions. **Table 1** summarizes the currently recognized screening strategies for several major cancer types (24–32).

**Table 1:** Current screening strategies for major cancers.

Cancer type	Screening strategy
Lung cancer (24)	Based on the NLST and NELSON trials, LDCT is the only modality proven to reduce lung-cancer-specific mortality and is recommended for high-risk individuals
Liver cancer (25)	Biannual liver ultrasonography combined with serum α-fetoprotein measurement is the cornerstone for early detection in high-risk patients, such as those with cirrhosis or chronic hepatitis B infection.
Prostate cancer (26–28)	Shared decision-making for PSA-based screening in men aged 50–69, with individual risk stratification (family history, African ancestry). Screening interval: every 2–4 years if PSA < 1 ng/mL. Avoid routine screening in men >70 or life expectancy <10–15 years.
Breast cancer (29)	Contemporary consensus guidelines recommend initiating a shared decision-making conversation about mammographic screening at age 40, balancing potential benefits against associated harms.
Colorectal cancer (30)	The recommended starting age for population-based screening has been lowered to 45 years.
Gastric cancer (31)	In high-incidence regions, biennial upper-gastroscopy or double-contrast barium radiography is advised for individuals ≥ 50 years.
Cervical cancer (32)	All individuals with a cervix aged 21–65 years should undergo cervical cancer screening at least every five years.

LDCT, low-dose computed tomography.

It is well known that most types of cancer can be prevented by adopting a healthy lifestyle, but there are still some cancers that cannot be prevented (33). Therefore, appropriate interventions for preventable cancers can yield substantial benefits, making the development of effective screening and prevention strategies a critical issue that needs to be addressed. Previous literature has systematically classified cancer risk factors into two major categories: “modifiable” and “unmodifiable”, and quantified their population attribution risks (33). Modifiable factors such as smoking, drinking, obesity, lack of exercise, poor diet, type 2 diabetes, hypertension and infections (such as HPV, HBV) can explain 30% to 70% of cancer cases. Active intervention of these factors can significantly decrease the lifetime cancer risk (34–37). Non-modifiable risk factors include increasing age, male sex, ethnic background, family history of cancer, and inherited high-penetrance mutations such as BRCA1/2 and CDKN2A. In addition, somatic mutations that accumulate during DNA replication also contribute to cancer susceptibility. These factors largely determine an individual’s inherent risk and are typically not preventable (33, 34, 36, 37).

The current traditional cancer awareness and screening models have three major limitations, and these limitations are particularly prominent among low-resource or marginalized populations. First, low participation rates; For lung cancer, a 2017 nationwide study revealed that just 3.9% of high-risk current or former smokers had undergone low-dose CT (LDCT) screening within the previous year (38).Even in organized national programs for screening breast cancer and cervical cancer, the overall population participation rate is still less than 50-60% (39–42). Participation rates were even lower for marginalized groups (rural, ethnic minorities, immigrants, people with low incomes or severe mental disorders) (42, 43).A meta-analysis incorporating 658 studies covering breast cancer, cervical cancer, lung cancer, colorectal cancer, gastric cancer, and prostate cancer also highlighted that barriers to cancer screening across multiple tumor types are complex, encompassing demographic and patient-level factors, socioeconomic factors, provider and community challenges, and access to healthcare. Screening rates for these cancers remain consistently low (44). Second, an equity gap persists, reflected in disparities across urban and rural areas, socioeconomic status, cultural backgrounds, and language, all of which contribute to significant inequalities in access to cancer screening. For example, the coverage rate of cervical cancer screening in rural China is approximately 25%, compared to over 35% in urban areas (39). Moreover, rural cases are often diagnosed at more advanced stages and are associated with higher mortality rates (45, 46). Furthermore, research indicates disparities in breast, colorectal, and lung cancer outcomes across different populations, particularly among underrepresented minorities and individuals with lower socioeconomic status. This underscores the significant reality of unequal access to screening (47). Third, the traditional “one-size-fits all” age or gender criteria cannot identify the true ultra-high-risk individuals, resulting in overscreening of low-risk populations and missed detection of high-risk populations (48, 49). Moreover, both the public and healthcare providers show limited acceptance of personalized screening based on genetics and lifestyle factors, expressing concerns about algorithm transparency, privacy, and fair resource allocation (50).

## 3 AI and digital technologies in cancer prevention and screening

### 3.1 AI for risk assessment and prediction

The field of oncology is experiencing rapid development in the application of big data and artificial intelligence. Recent advances in artificial intelligence have facilitated the integration and analysis of multi-modal data across scales. These methods are increasingly being applied to extract insights from large and complex datasets, with several studies demonstrating their utility in oncology settings. The application of machine learning in the medical field, particularly in oncology, has become increasingly widespread in recent years. The complexity and heterogeneity of cancer provide a large amount of multimodal data, creating application conditions for machine learning.

Artificial intelligence has shown great promise in cancer risk prediction, encompassing several key approaches. One major area involves the use of machine learning models for personalized risk stratification based on individual-level features such as genetics, lifestyle, and clinical history. Additionally, the integration of electronic health records (EHRs) and large-scale population data enables the identification of high-risk individuals and groups through pattern recognition and predictive analytics. These approaches allow for earlier detection and targeted interventions, ultimately improving cancer prevention strategies at both the individual and population levels.

Beyond individual prediction, several population-level studies have demonstrated that AI-enabled screening programs can enhance early detection and potentially reduce cancer mortality through more efficient risk-based resource allocation. For example, regional modeling analyses have shown that AI-assisted mammography or LDCT triage systems can maintain or improve diagnostic sensitivity while reducing screening workload and costs (51, 52). Moreover, AI-driven digital outreach tools have been used to improve participation rates and screening equity among underserved populations in low-resource settings. By combining precision risk prediction with public health implementation, AI contributes not only to personalized prevention but also to population-level benefits—improving efficiency, equity, and cost-effectiveness across cancer screening systems.

**Figure 2:**
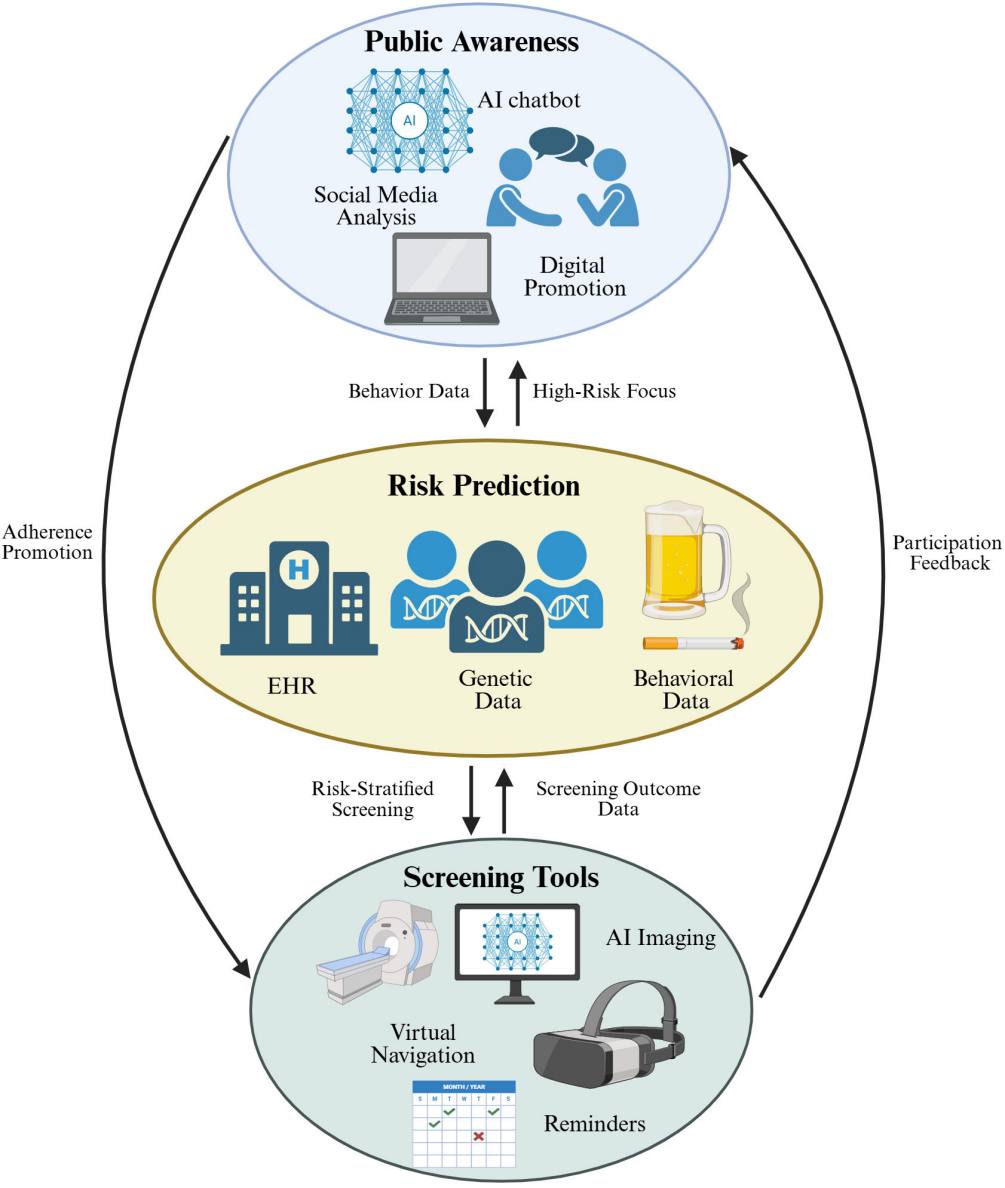
A comprehensive overview of AI applications in cancer detection and screening. The diagram consists of three core links with mutual feedback: (1) Top: AI-driven tools including AI chatbot, social media analysis, and digital promotion to enhance public cancer cognition; (2) Middle: AI models integrating electronic health records, genetic data, and behavioral data for personalized cancer risk assessment; (3) Bottom: AI-enabled tools such as AI imaging, virtual navigation, and reminders to optimize screening management. Black arrows represent mutual feedback loops, indicating data flow and interaction between different links.

Artificial intelligence contributes to cancer prevention and screening through a multi-layered approach, ranging from enhancing public awareness to refining risk prediction and supporting clinical screening tools. These components are not isolated; rather, they form an interactive ecosystem in which information and feedback flow bidirectionally. To provide a comprehensive overview of this integrated framework, we illustrate the landscape of AI applications across public awareness, risk prediction, and screening management (**Figure 2**).

Johnson et al. have developed an AI tool that innovatively combines genomics with computational models to create a digital simulation system similar to weather forecasts. This system can predict the changes in cell activity in tissues over time, simulate cancer growth, immune responses, and treatment effects, and facilitate personalized treatment. Moreover, the tool is open source and cross-disciplinary applicable (53). Placido applied an artificial intelligence approach to the collected data by training a machine learning model on sequences of disease codes derived from clinical histories and evaluating its ability to predict cancer occurrence within incremental time windows. This approach not only predicts the likelihood of cancer occurrence but also provides dynamic risk assessments at incremental time intervals following the initial prediction (54). All of these findings highlight the potential of artificial intelligence to leverage written medical records and multi-omics data to predict cancer occurrence in large populations.

However, many AI models, especially deep learning models, operate as “black boxes,” making it difficult for clinicians to understand the rationale behind predictions and for patients to fully trust AI-assisted decisions (55, 56). This limitation should be given due attention in subsequent clinical applications.

### 3.2 AI in imaging and screening tools

Radiomics is an emerging subfield of medical imaging that integrates medical imaging, bioinformatics, data science, and statistics. It digitally decodes medical images [such as computed tomography (CT), magnetic resonance imaging (MRI), and positron emission tomography (PET), among others] into a large number of quantitative features, including shape, size, and texture patterns (57, 58). AI is good at exploring the nonlinear and high-dimensional relationships between radiomics features (58, 59). Massive datasets also provide rich materials for the training of AI models.

The AI model PLAN-B-DF, developed by Hyunjae Shin’s team, significantly improved the accuracy of liver cancer risk prediction in patients with chronic hepatitis B by integrating CT imaging markers (e.g., visceral fat ratio, muscle density) with dynamic clinical physiological indicators. The model demonstrated high predictive performance, with a C-index of 0.91 in internal validation and 0.89 in external validation. It enabled precise individualized risk stratification, with a 10-year incidence rate of 46.2% in the high-risk group compared to 0% in the extremely low-risk group. This innovative approach overcomes the limitations of traditional models that rely solely on clinical variables and establishes a new paradigm for liver cancer surveillance using quantifiable imaging biomarkers (60). In a multicenter study, pretreatment FDG-PET/CT images were analyzed using machine learning to predict lung cancer progression risk and overall survival (OS). This study reviewed 965 patients (1168 nodules) from three institutions. Convolutional neural networks (CNNs) were used to predict tumor progression, combined with random survival forest (RSF) models and radiomics features to predict survival, and compared the performance of single-modality (PET or CT) and ensemble models. Finally, the deep learning model based on FDG-PET/CT has a high value in the prognosis evaluation of lung cancer (61). For the early diagnosis of gallbladder cancer, Xiang et al. developed a deep learning model based on the ResNet50 network. This model can distinguish gallbladder cancer from benign gallbladder lesions through contrast-enhanced CT images, and both the AUC value and sensitivity have reached a relatively high level (62).In addition, machine learning has been applied to predict the risk of breast and prostate cancers using modalities such as mammography and MRI.

Computer-aided diagnosis (CAD) is an interdisciplinary technology that uses computers to analyze medical images or pathological data to assist clinicians in making relevant diagnoses. At present, CAD system is also widely used in clinical practice. For example, it has made great breakthroughs in the diagnosis of benign and malignant lung nodules (63), breast cancer (64) and brain tumors (65), which greatly improves the efficiency of early diagnosis.

This study briefly summarizes the research on AI-assisted imaging for the screening and diagnosis of various types of cancer (**Table 2**) (66–100).In conclusion, the integration of artificial intelligence with deep learning–based risk prediction models enables the design of personalized screening strategies and substantially improves the detection rate of early-stage cancers. Designing more technologies for more cancer types to improve the overall diagnostic efficiency will become a new direction for future development.

**Table 2:** AI-assisted imaging screening strategies for major cancers.

Cancer type	AI-assisted imaging screening and diagnosis
Lung cancer (66–70)	Deep learning models based on low-dose CT can enable individualized risk prediction and assist in nodule interpretation, demonstrating the potential to improve sensitivity and reduce false positives.
Liver cancer (71–75)	AI models can detect and classify liver lesions using ultrasound, CEUS, and multimodal imaging. While some multicenter studies have demonstrated their superiority over traditional methods, their actual clinical value in the long-term screening of high-risk populations is still being explored.
Prostate cancer (76–79)	Deep learning models analyze magnetic resonance imaging (MRI) and pathology data to improve the accuracy of prostate cancer detection and standardize diagnostic procedures. For patients with localized prostate cancer, AI-assisted risk stratification offers more precise treatment recommendations based on characteristics such as PSA levels and Gleason scores. Multimodal deep learning architectures integrate digital pathology images with clinical data to predict long-term outcomes, guiding treatment decisions.
Breast cancer (80–84)	Multiple large-scale retrospective and randomized trials have confirmed that AI can significantly reduce the workload of radiologists while maintaining or improving cancer detection rates in mammography screening. However, key endpoints such as “interval cancer rates” still require long-term follow-up to validate.
Colorectal cancer (85–90)	Multiple randomized controlled trials (RCTs) and real-world studies have demonstrated that AI-assisted colonoscopy (CADe) can significantly improve adenoma detection rates and reduce false negative rates. Some guidelines have already recommended its use, but there are discrepancies among different studies regarding the extent to which it improves the overall detection rate of precancerous lesions.
Gastric cancer (87–92)	Convolutional neural networks demonstrate high sensitivity in the early detection of gastric cancer in endoscopic images and can assist in determining the depth of invasion and lesion boundaries.
Cervical cancer (93–96)	Deep learning-based automated visual assessment (AVE) can accurately identify high-grade lesions in cervical images and has been validated for feasibility in smartphone applications in low-resource areas, but it is still in the early stages of clinical validation and promotion.

MRI, magnetic resonance imaging; RC, randomized controlled trials; CADe, AI-assisted colonoscopy; AVE, automated visual assessment.

It should also be noted that models trained on specific datasets may perform less accurately on different populations, rare cancer subtypes, or new imaging protocols, reflecting the problem of dataset shift (101, 102). This remains an important challenge that requires further research and optimization in AI imaging applications.

### 3.3 AI chatbots and large language models for public awareness

With the rapid advancement of artificial intelligence technology, various AI models, both domestic and international, such as ChatGPT and DeepSeek, have been widely adopted in daily life. The growing accessibility and performance of such technologies have not only transformed communication, education, and business, but have also opened new opportunities for innovation in healthcare.

Scientists at Harvard Medical School designed a multifunctional, ChatGPT-like AI model named CHIEF, which is capable of performing a range of diagnostic tasks for a variety of cancers. The new model can perform a wide range of tasks and has been tested on 19 cancer types, giving it similar flexibility to LLMs such as ChatGPT (103). CHIEF has achieved an accuracy rate of nearly 94% in cancer detection, significantly outperforming current artificial intelligence methods. Columbia University and Cedars-Sinai Medical Center jointly utilized a LLM to generate embeddings of disease names in electronic health records (EHR) and integrated them into the Transformer model to mine potential signals in EHR, achieving early warning for pancreatic cancer patients. It significantly improved the predictive performance of pancreatic cancer at 6–12 months and earlier stages. This method is independent of traditional risk factors, with a PPV as high as 0.141, providing a new idea for early screening (104). Haver’s study demonstrated that ChatGPT holds considerable potential for automating the delivery of patient education on breast cancer prevention and screening (105). Recent research results also show that the application of LLM has achieved significant improvements in all aspects of breast cancer management. The efficiency of diagnosis and treatment has increased by 35%, the clinical trust and reliability of the system have improved by 30%, and the quality of patient education and information has improved by 20% (106).

However, LLMs for tumor diagnosis still face substantial challenges and are not yet capable of making fully accurate and comprehensive diagnostic decisions. They are prone to hallucinations and incomplete knowledge. Nevertheless, LLMs can already serve as valuable tools to support the screening and analysis of large biomedical datasets. In the future, LLMs should undergo further training, rigorous validation, and optimization to achieve higher accuracy and greater reliability.

### 3.4 Multi-cancer early detection strategy combining whole-genome and AI algorithms

Early cancer diagnosis is crucial for improving survival rates. However, current screening methods are limited in the types of cancer they cover. They are also invasive and have poor compliance, which makes it difficult to meet the clinical demand for the early screening of multiple types of cancer. Due to their non-invasive nature and potential for pan-cancer coverage, molecular markers based on circulating cell-free DNA (cfDNA) in plasma have become a hot topic of research. A recent study by the Bao team has, for the first time, systematically extracted multidimensional information from cfDNA and used AI to integrate multidimensional data for cancer identification, covering 13 types of cancer, some of which do not currently have standard screening methods (107). The study demonstrated high sensitivity and specificity across training sets, validation sets, and asymptomatic populations, indicating its high sensitivity to early cancer signals. It is particularly suitable for cancer types not covered by traditional screening methods (such as pancreatic cancer and liver cancer) and can provide clear direction for subsequent clinical interventions. This breakthrough also highlights the immense potential of AI in the precise early screening of tumors. Similar efforts have been reported by other groups; for example, Cohen et al. developed the CancerSEEK assay for multi-cancer early detection using cfDNA and protein biomarkers, showing promising performance for several cancer types (108), and Lennon et al. applied machine learning to methylation profiles of cfDNA for pan-cancer detection (109). These studies collectively underscore the potential of AI-assisted cfDNA analysis in precise early cancer screening, particularly for cancers not covered by traditional screening, such as pancreatic and liver cancer, and provide clear directions for subsequent clinical interventions.

### 3.5 AI-driven personalized screening pathway

**Figure 3:**
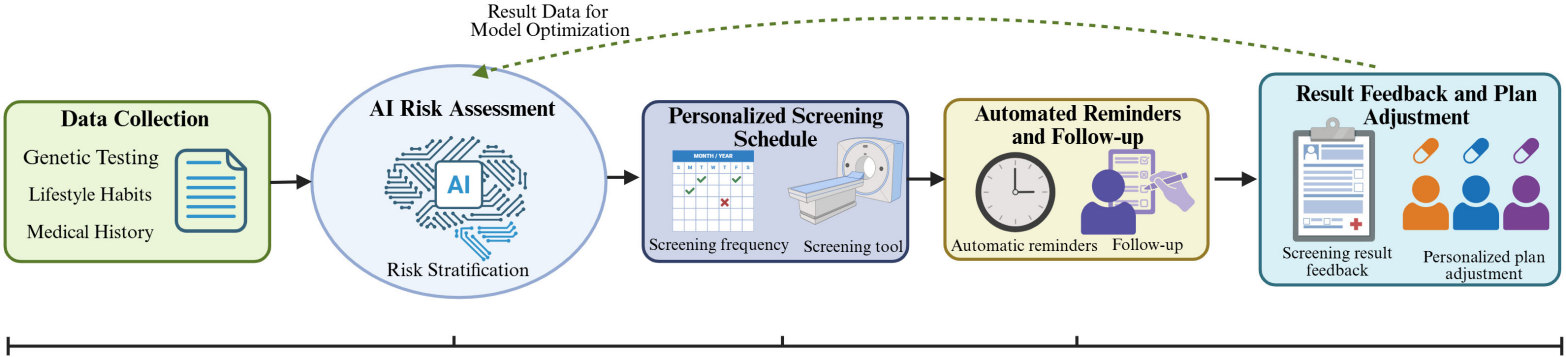
AI-driven personalized cancer screening journey map. The map follows a patient-centric sequential process with closed-loop feedback: (1) Data Collection: Integrating genetic testing, lifestyle habits, and medical history to provide basic data support; (2) AI Risk Assessment: Using AI models to conduct risk stratification based on collected data; (3) Personalized Screening Schedule: Developing targeted screening frequency and items according to risk levels; (4) Automated Reminders and Follow-up: Improving screening adherence through AI-enabled reminders and follow-up management; (5) Result Feedback and Plan Adjustment: Feeding back screening results to optimize AI risk assessment models and adjust subsequent personalized plans.

To further illustrate the implementation framework of AI in personalized cancer screening, **Figure 3** presents a patient journey map that outlines the sequential process from data input to closed-loop optimization. This map systematically integrates five key links, including data collection (genetic testing, lifestyle habits, and medical history), AI-driven risk assessment, development of personalized screening schedules, automated reminders and follow-up, and result feedback for plan adjustment, thereby highlighting the continuous and adaptive nature of AI-enabled personalized screening.

## 4 Strategies to improve awareness of cancer risk factors

Raising patient awareness of cancer risk factors is a critical component of effective cancer prevention and early detection. Patient health education is an integral component of medical care, permeating the entire healthcare process—from patient admission and discharge to post-discharge rehabilitation and follow-up. Through health education, patients can gain a better understanding of their condition and treatment plans, and actively adhere to healthcare regimens, thereby enhancing treatment adherence and improving rehabilitation outcomes. Patient health education should not only provide general information but also emphasize individualized risk profiles, including genetic predisposition, lifestyle, and environmental exposures. Advances in artificial intelligence and big data have facilitated the development of more personalized and targeted approaches to health education, which may enhance the effectiveness of cancer prevention and screening programs. Significant improvements will be made in addressing the numerous shortcomings in patient health education, including content, format, assessment, and resource allocation. Emerging technologies such as artificial intelligence are facilitating the transformation of traditional health education models toward more patient-centered, integrated, and continuous approaches.

AI can assist in identifying individuals at higher risk of specific cancers by analyzing genetic, clinical, and lifestyle data, thereby enabling personalized risk communication. For example, AI-driven platforms can integrate electronic health records, family history, and behavioral data to generate individualized cancer risk scores and provide tailored recommendations for screening or lifestyle modification (110). AI can also help users identify potential health risks and provide customized health recommendations through accurate analysis of health data. For example, smart health devices monitor physiological data such as heart rate, blood sugar, and body temperature, analyze users’ health conditions in real time, and provide timely health feedback. These devices not only perform daily monitoring but also analyze users’ health trends through long-term data accumulation, providing a scientific basis for health management (111). For women with a family history of breast cancer, AI can use genetic and lifestyle information to create a schedule for regular breast exams and send reminders. Additionally, AI can develop personalized health intervention plans based on users’ specific circumstances, helping them better manage their health. In light of the widespread adoption of social media, healthcare professionals can collaborate with engineers to develop AI-driven health education platforms that deliver personalized recommendations for cancer prevention, early screening, and healthy lifestyle choices. These platforms can respond to users’ specific inquiries regarding cancer risk, family history, or related symptoms, and provide tailored, evidence-based guidance and information.

## 5 AI for enhancing risk screening and management

Currently, AI can increase the participation rate of target groups in examinations or trials through precise recruitment, personalized reminders, mobile applications, and chatbots. For example, AI can automatically identify people who meet the screening criteria but have not yet made an appointment from electronic medical records and send personalized invitation text messages, thereby significantly increasing the appointment rate and attendance rate (112). Traditionally, patient screening for clinical trials has been a time-consuming manual process involving multiple eligibility checkpoints, including medical record reviews. Artificial intelligence is changing this by automatically collecting, aggregating, standardizing, and analyzing patient data and medical records to make potential patients eligible for specific trial criteria or qualify them for trials they have expressed interest in.

At the 2024 American Society of Clinical Oncology (ASCO) Annual Meeting, Dr. Alyson B. Moadel of the Einstein College of Medicine at Montefiore presented a study on artificial intelligence. The results showed that AI-based patient navigation tools can effectively help patients who failed to keep their initial colonoscopy appointments, significantly improving their completion rates (113).

Combining AI with medical insurance and resource allocation optimization to screen cancer patients is an important area of exploration in the medical field today. The integration of AI with healthcare insurance and resource allocation optimization has brought significant changes to cancer screening. Through technological innovation, screening costs have been reduced and efficiency improved, while resource allocation has been optimized, benefiting more patients. In the future, with the continuous advancement of technology and the improvement of policies, AI will play an even greater role in cancer prevention and control, helping to achieve the goal of “early screening and early treatment.”

## 6 Future directions of AI for risk awareness and screening management

As described in the previous sections, artificial intelligence has been widely applied in cancer prevention and screening. Although significant progress has been made, its application in precision medicine still faces many challenges.

There are currently several major issues. First, there is insufficient data standardization and interoperability. Medical data formats are inconsistent, and labeling is not standardized. This affects the training and generalization capabilities of AI models (114, 115). Such heterogeneity reduces model generalizability and hinders multicenter validation. To address this, initiatives such as the Cancer Imaging Archive (TCIA) have promoted standardized imaging and annotation protocols to improve data consistency and accessibility (116). Second, model generalizability remains a critical concern. AI systems trained on specific populations or single-center datasets may not perform equally well in different healthcare settings or among diverse ethnic groups (117, 118). Third, data sharing between medical and research institutions and enterprises is difficult, which limits the scale and diversity of AI model training data. This makes it difficult to comprehensively cover the characteristics and mutations of various types of cancer (119–121).Fourth, the decision-making process of deep learning models is complex and difficult to explain intuitively, resulting in low trust in the results among doctors and patients. In clinical applications, it is necessary to clarify the basis for the model’s judgments and potential risks (122, 123). Additionally, AI models tend to perform well when trained on specific datasets but may struggle when confronted with different populations, cancer types, or rare cases. For instance, while some models perform well in screening for common cancers, their accuracy declines when it comes to detecting rare cancers or special subtypes. Finally, in terms of privacy and security risks, cancer screening involves a large amount of sensitive personal information, and data breaches could lead to patient privacy violations. At the same time, the security of AI systems must be ensured to prevent malicious attacks and data tampering (124–126).

AI also faces some challenges in its clinical application for early cancer screening. For example, although AI models perform well in certain tasks, there is still a risk of misdiagnosis. False positives may lead to patients undergoing unnecessary tests and treatments, increasing the burden on healthcare systems; false negatives may delay diagnosis (127–129).

In addition to technical and clinical challenges, the ethical and governance context of AI applications in cancer screening is receiving increasing attention. Recent international and national frameworks have provided guiding principles to ensure that AI technologies are developed and used responsibly. For example, the World Health Organization’s 2024 “Ethics and Governance of Artificial Intelligence for Health” report emphasizes human oversight, accountability, transparency, and equitable access as core values for AI in healthcare (130). Similarly, the European Union Artificial Intelligence Act (2024) classifies medical AI tools as high-risk systems and requires robust validation, post-market monitoring, and explainability to ensure patient safety (131).These frameworks collectively aim to balance innovation with protection—ensuring that data-driven cancer screening systems are ethical, transparent, and socially trustworthy. Incorporating such principles into AI system design and clinical implementation can enhance patient confidence, promote international collaboration, and accelerate the safe translation of AI tools into population-level cancer prevention and screening programs.

In the future, measures such as data standardization, model interpretability research, interdisciplinary collaboration, and policy support are needed to promote the safe and effective application of AI technology in cancer prevention and control. Additionally, there is currently limited real-world research data on AI. In the future, large-scale, prospective cohort studies with large sample sizes should be conducted to evaluate the effectiveness of different AI tools in the early detection of cancer. At the same time, AI can be used to develop personalized follow-up plans for patients, enabling more effective monitoring of cancer changes, improving patient prognosis, and guiding early intervention and medication. As AI technology continues to advance and receive policy support, we believe that artificial and AI-assisted decision-making will become a new model for efficient screening and diagnosis.

## 7 Conclusion

“Early detection, early diagnosis, and early treatment” is globally recognized as the most effective strategy for cancer prevention and control, significantly improving patient survival rates and reducing social healthcare expenditures.

Emerging evidence suggests AI has the potential to influence cancer screening workflows. With technologies such as machine learning, big data, and LLMs, AI shows great potential in risk prediction, individualized screening plan development, and health communication based on individual characteristics. These innovations have the potential to solve long-standing problems, such as low screening participation rates, unequal access, and delayed early detection.

However, realizing the full potential of AI in this field depends not only on technological advances, but also on the establishment of sound data privacy protection, algorithm transparency, and ethical regulatory frameworks.Thorough validation of AI tools in real-world clinical and population-based settings is essential to assess their effectiveness and generalizability across diverse populations and cancer types. Integrating AI into national and regional screening programs can optimize coverage, efficiency, and equity, while cost–benefit analyses help evaluate financial sustainability and inform resource allocation. The development of open, shared databases and continuous evaluation of ethical, legal, and social implications further support reproducible research, continuous model improvement, and responsible implementation.Looking forward, continued progress in this field will depend on multidisciplinary collaboration. Clinicians, artificial intelligence researchers, public health experts, and policymakers must work together to design, evaluate, and promote solutions that improve the accuracy and efficiency of screening while fostering trust and inclusivity. Creating an open, shared public database is also essential to advancing related research. Through this collaborative approach, AI will become a transformative force in reducing the global burden of cancer by bringing timely prevention and screening interventions to more people.

## References

[1] Filho AM Laversanne M Ferlay J Colombet M Piñeros M Znaor A . The GLOBOCAN 2022 cancer estimates: Data sources, methods, and a snapshot of the cancer burden worldwide. Int J Cancer. (2025) 156:1336–46. doi: 10.1002/ijc.35278, PMID: 39688499

[2] Siegel RL Kratzer TB Giaquinto AN Sung H Jemal A . Cancer statistics, 2025. CA Cancer J Clin. (2025) 75:10–45. doi: 10.3322/caac.21871, PMID: 39817679 PMC11745215

[3] Miller KD Nogueira L Devasia T Mariotto AB Yabroff KR Jemal A . Cancer treatment and survivorship statistics, 2022. CA Cancer J Clin. (2022) 72:409–36. doi: 10.3322/caac.21731, PMID: 35736631

[4] Allemani C Matsuda T Di Carlo V Harewood R Matz M Nikšić M . Global surveillance of trends in cancer survival 2000-14 (CONCORD-3): analysis of individual records for 37 513 025 patients diagnosed with one of 18 cancers from 322 population-based registries in 71 countries. Lancet. (2018) 391:1023–75. doi: 10.1016/S0140-6736(17)33326-3, PMID: 29395269 PMC5879496

[5] Crosby D Bhatia S Brindle KM Coussens LM Dive C Emberton M . Early detection of cancer. Science. (2022) 375:eaay9040. doi: 10.1126/science.aay9040, PMID: 35298272

[6] Goddard KAB Feuer EJ Mandelblatt JS Meza R Holford TR Jeon J . Estimation of cancer deaths averted from prevention, screening, and treatment efforts, 1975-2020. JAMA Oncol. (2025) 11:162–7. doi: 10.1001/jamaoncol.2024.5381, PMID: 39636625 PMC11622066

[7] Lawrence R Watters M Davies CR Pantel K Lu YJ . Circulating tumour cells for early detection of clinically relevant cancer. Nat Rev Clin Oncol. (2023) 20:487–500. doi: 10.1038/s41571-023-00781-y, PMID: 37268719 PMC10237083

[8] Aberle DR Adams AM Berg CD Black WC Clapp JD Fagerstrom RM . Reduced lung-cancer mortality with low-dose computed tomographic screening. N Engl J Med. (2011) 365:395–409. doi: 10.1056/NEJMoa1102873, PMID: 21714641 PMC4356534

[9] Mittra I Mishra GA Dikshit RP Gupta S Kulkarni VY Shaikh HKA . Effect of screening by clinical breast examination on breast cancer incidence and mortality after 20 years: prospective, cluster randomised controlled trial in Mumbai. BMJ. (2021) 372:n256. doi: 10.1136/bmj.n256, PMID: 33627312 PMC7903383

[10] Xia C Li H Xu Y Guo G Yu X Wang W . Effect of an endoscopy screening on upper gastrointestinal cancer mortality: A community-based multicenter cluster randomized clinical trial. Gastroenterology. (2025) 168:725–40. doi: 10.1053/j.gastro.2024.11.025, PMID: 39706350

[11] Rumgay H Shield K Charvat H Ferrari P Sornpaisarn B Obot I . Global burden of cancer in 2020 attributable to alcohol consumption: a population-based study. Lancet Oncol. (2021) 22:1071–80. doi: 10.1016/S1470-2045(21)00279-5, PMID: 34270924 PMC8324483

[12] Singal AG Kanwal F Llovet JM . Global trends in hepatocellular carcinoma epidemiology: implications for screening, prevention and therapy. Nat Rev Clin Oncol. (2023) 20:864–84. doi: 10.1038/s41571-023-00825-3, PMID: 37884736

[13] Kocarnik JM Compton K Dean FE Fu W Gaw BL Harvey JD . Cancer incidence, mortality, years of life lost, years lived with disability, and disability-adjusted life years for 29 cancer groups from 2010 to 2019: A systematic analysis for the global burden of disease study 2019. JAMA Oncol. (2022) 8:420–44. doi: 10.1001/jamaoncol.2021.6987, PMID: 34967848 PMC8719276

[14] Soerjomataram I Bray F . Planning for tomorrow: global cancer incidence and the role of prevention 2020-2070. Nat Rev Clin Oncol. (2021) 18:663–72. doi: 10.1038/s41571-021-00514-z, PMID: 34079102

[15] GBD 2019 Cancer Risk Factors Collaborators The global burden of cancer attributable to risk factors, 2010-19: a systematic analysis for the Global Burden of Disease Study 2019. Lancet. (2022) 400:563–91. doi: 10.1016/S0140-6736(22)01438-6, PMID: 35988567 PMC9395583

[16] Hamet P Tremblay J . Artificial intelligence in medicine. Metabolism. (2017) 69s:S36–s40. doi: 10.1016/j.metabol.2017.01.011, PMID: 28126242

[17] Xing Z Chen J Pan L Huang D Qiu Y Sheng C . Enhanced detection of prostate cancer lesions on biparametric MRI using artificial intelligence: A multicenter, fully-crossed, multi-reader multi-case trial. Acad Radiol. (2025) 32:5954–63. doi: 10.1016/j.acra.2025.06.038, PMID: 40651921

[18] Marcu LG Marcu DC . Examining the role of AI in cancer imaging through the lens of clinical studies. Health Technol. (2025) 15:1065–74. doi: 10.1007/s12553-025-01019-w

[19] Jafree SR Bukhari N Muzamill A Tasneem F Fischer F . Digital health literacy intervention to support maternal, child and family health in primary healthcare settings of Pakistan during the age of coronavirus: study protocol for a randomised controlled trial. BMJ Open. (2021) 11:e045163. doi: 10.1136/bmjopen-2020-045163, PMID: 33653760 PMC7929637

[20] Kung TH Cheatham M Medenilla A Sillos C De Leon L Elepaño C . Performance of ChatGPT on USMLE: Potential for AI-assisted medical education using large language models. PloS Digital Health. (2023) 2:e0000198. doi: 10.1371/journal.pdig.0000198, PMID: 36812645 PMC9931230

[21] Jiang LY Liu XC Nejatian NP Nasir-Moin M Wang D Abidin A . Health system-scale language models are all-purpose prediction engines. Nature. (2023) 619:357–62. doi: 10.1038/s41586-023-06160-y, PMID: 37286606 PMC10338337

[22] Hao Y Qiu Z Holmes J Löckenhoff CE Liu W Ghassemi M . Large language model integrations in cancer decision-making: a systematic review and meta-analysis. NPJ Digital Med. (2025) 8:450. doi: 10.1038/s41746-025-01824-7, PMID: 40676129 PMC12271406

[23] Rintala S Dahlstrom KR Franco EL Louvanto K . A synthesis of evidence for cancer-specific screening interventions: A Preventive Medicine Golden Jubilee Review. Prev Med. (2023) 167:107395. doi: 10.1016/j.ypmed.2022.107395, PMID: 36565859

[24] de Koning HJ van der Aalst CM de Jong PA . Reduced lung-cancer mortality with volume CT screening in a randomized trial. N Engl J Med. (2020) 382:503–13. doi: 10.1056/NEJMoa1911793, PMID: 31995683

[25] Hwang SY Danpanichkul P Agopian V Mehta N Parikh ND Abou-Alfa GK . Hepatocellular carcinoma: updates on epidemiology, surveillance, diagnosis and treatment. Clin Mol Hepatol. (2025) 31:S228–s54. doi: 10.3350/cmh.2024.0824, PMID: 39722614 PMC11925437

[26] Wei JT Barocas D Carlsson S Coakley F Eggener S Etzioni R . Early detection of prostate cancer: AUA/SUO guideline part I: prostate cancer screening. J Urol. (2023) 210:46–53. doi: 10.1097/JU.0000000000003491, PMID: 37096582 PMC11060750

[27] Grossman DC Curry SJ Owens DK Bibbins-Domingo K Caughey AB Davidson KW . Screening for prostate cancer: US preventive services task force recommendation statement. JAMA. (2018) 319:1901–13. doi: 10.1001/jama.2018.3710, PMID: 29801017

[28] Moses KA Sprenkle PC Bahler C Box G Carlsson SV Catalona WJ . NCCN guidelines^®^ Insights: prostate cancer early detection, version 1.2023. J Natl Compr Cancer Netw: JNCCN. (2023) 21:236–46. doi: 10.6004/jnccn.2023.0014, PMID: 36898362

[29] Rahman WT Helvie MA . Breast cancer screening in average and high-risk women. Best Pract Res Clin Obstet Gynaecol. (2022) 83:3–14. doi: 10.1016/j.bpobgyn.2021.11.007, PMID: 34903436

[30] Bibbins-Domingo K Grossman DC Curry SJ Davidson KW Epling JW Jr García FAR . Screening for colorectal cancer: US preventive services task force recommendation statement. JAMA. (2021) 325:1965–77. doi: 10.1001/jama.2021.6238, PMID: 34003218

[31] Kusano C Ishibashi F Ichita C Gotoda T . Current status of gastric cancer screening and future perspectives. DEN Open. (2026) 6:e70148. doi: 10.1002/deo2.70148, PMID: 40433232 PMC12106035

[32] Voelker RA . Cervical cancer screening. JAMA. (2023) 330:2030. doi: 10.1001/jama.2023.21987, PMID: 37889510

[33] Marino P Mininni M Deiana G Marino G Divella R Bochicchio I . Healthy lifestyle and cancer risk: modifiable risk factors to prevent cancer. Nutrients. (2024) 16:800. doi: 10.3390/nu16060800, PMID: 38542712 PMC10974142

[34] Olakowski M Bułdak Ł . Modifiable and non-modifiable risk factors for the development of non-hereditary pancreatic cancer. Med (Kaunas Lithuania). (2022) 58. doi: 10.3390/medicina58080978, PMID: 35893093 PMC9394367

[35] Gelfond J Al-Bayati O Kabra A Iffrig K Kaushik D Liss MA . Modifiable risk factors to reduce renal cell carcinoma incidence: Insight from the PLCO trial. Urologic Oncol. (2018) 36:340.e1–.e6. doi: 10.1016/j.urolonc.2018.04.011, PMID: 29779672 PMC11697291

[36] Midha S Chawla S Garg PK . Modifiable and non-modifiable risk factors for pancreatic cancer: A review. Cancer Lett. (2016) 381:269–77. doi: 10.1016/j.canlet.2016.07.022, PMID: 27461582

[37] Xu H Xu B . Breast cancer: Epidemiology, risk factors and screening. Chin J Cancer Res. (2023) 35:565–83. doi: 10.21147/j.issn.1000-9604.2023.06.02, PMID: 38204449 PMC10774137

[38] Jemal A Fedewa SA . Lung cancer screening with low-dose computed tomography in the United States-2010 to 2015. JAMA Oncol. (2017) 3:1278–81. doi: 10.1001/jamaoncol.2016.6416, PMID: 28152136 PMC5824282

[39] Zhang M Wang L Zhang X Li C Zhao Z Yu M . Cervical cancer screening rates among chinese women - China, 2023-2024. China CDC Weekly. (2025) 7:321–6. doi: 10.46234/ccdcw2025.052, PMID: 40225781 PMC11982917

[40] Lee K Suh M Choi KS . Current status of the national cancer screening program in korea: history, achievements, and future directions. J Prev Med Public Health = Yebang Uihakhoe Chi. (2025) 58:337–47. doi: 10.3961/jpmph.25.268, PMID: 40575987 PMC12332392

[41] Institut National du Cancer . Panorama des cancers en France, edition 2023. New York, NY, USA: Technical report, Institut National du Cancer (2023).

[42] Fuzzell LN Perkins RB Christy SM Lake PW Vadaparampil ST . Cervical cancer screening in the United States: Challenges and potential solutions for underscreened groups. Prev Med. (2021) 144:106400. doi: 10.1016/j.ypmed.2020.106400, PMID: 33388330

[43] Yan H Wang Q Dang L Duan X Bai Z Feng Y . Implementation and maintenance of breast cancer screening among Chinese rural women: a mixed-methods evaluation based on RE-AIM framework. BMC Public Health. (2025) 25:2502. doi: 10.1186/s12889-025-23679-z, PMID: 40681982 PMC12273151

[44] Aguiar-Ibáñez R Mbous Y Sharma S Chakali R Chawla E . Barriers to cancer screening uptake and approaches to overcome them: a systematic literature review. Front Oncol. (2025) 15:1575820. doi: 10.3389/fonc.2025.1575820, PMID: 40842583 PMC12364675

[45] Liu P Zhu Y Zhou H Li R . Trends and urban-rural disparities in cervical cancer epidemiology in China, 2005-2018. Sci Rep. (2025) 15:25021. doi: 10.1038/s41598-025-09004-z, PMID: 40645988 PMC12254349

[46] Alshammari AH Ishii H Hirotsu T Hatakeyama H Morishita M di Luccio E . Bridging the gap in cervical cancer screening for underserved communities: MCED and the promise of future technologies. Front Oncol. (2024) 14:1407008. doi: 10.3389/fonc.2024.1407008, PMID: 39135996 PMC11317246

[47] Berland LL Monticciolo DL Flores EJ Malak SF Yee J Dyer DS . Relationships between health care disparities and coverage policies for breast, colon, and lung cancer screening. J Am Coll Radiol: JACR. (2019) 16:580–5. doi: 10.1016/j.jacr.2018.12.025, PMID: 30947890

[48] Li C Cheng B Li J Xiong S Fu W Jiang Y . Non-risk-based lung cancer screening with low-dose computed tomography. JAMA. (2025) 333:2108–10. doi: 10.1001/jama.2025.4017, PMID: 40257816 PMC12012712

[49] Knoppers BM Bernier A Granados Moreno P Pashayan N . Of screening, stratification, and scores. J Personalized Med. (2021) 11. doi: 10.3390/jpm11080736, PMID: 34442379 PMC8398020

[50] Tan NQP Nargund RS Douglas EE Lopez-Olivo MA Resong PJ Ishizawa S . Acceptability and perceptions of personalised risk-based cancer screening among health-care professionals and the general public: a systematic review and meta-analysis. Lancet Public Health. (2025) 10:e85–96. doi: 10.1016/S2468-2667(24)00278-0, PMID: 39909697 PMC11817692

[51] Eisemann N Bunk S Mukama T Baltus H Elsner SA Gomille T . Nationwide real-world implementation of AI for cancer detection in population-based mammography screening. Nat Med. (2025) 31:917–24. doi: 10.1038/s41591-024-03408-6, PMID: 39775040 PMC11922743

[52] Ziegelmayer S Graf M Makowski M Gawlitza J Gassert F . Cost-effectiveness of artificial intelligence support in computed tomography-based lung cancer screening. Cancers. (2022) 14:4711–4733.e37. doi: 10.3390/cancers14071729, PMID: 35406501 PMC8997030

[53] Johnson JAI Bergman DR Rocha HL Zhou DL Cramer E Mclean IC . Human interpretable grammar encodes multicellular systems biology models to democratize virtual cell laboratories. Cell. (2025) 188:4711–33.e37. doi: 10.1016/j.cell.2025.06.048, PMID: 40713951 PMC13012569

[54] Placido D Yuan B Hjaltelin JX Zheng C Haue AD Chmura PJ . A deep learning algorithm to predict risk of pancreatic cancer from disease trajectories. Nat Med. (2023) 29:1113–22. doi: 10.1038/s41591-023-02332-5, PMID: 37156936 PMC10202814

[55] Liu X Faes L Kale AU Wagner SK Fu DJ Bruynseels A . A comparison of deep learning performance against health-care professionals in detecting diseases from medical imaging: a systematic review and meta-analysis. Lancet Digital Health. (2019) 1:e271–e97. doi: 10.1016/S2589-7500(19)30123-2, PMID: 33323251

[56] London AJ . Artificial intelligence and black-box medical decisions: accuracy versus explainability. Hastings Center Rep. (2019) 49:15–21. doi: 10.1002/hast.973, PMID: 30790315

[57] Bi WL Hosny A Schabath MB Giger ML Birkbak NJ Mehrtash A . Artificial intelligence in cancer imaging: Clinical challenges and applications. CA Cancer J Clin. (2019) 69:127–57. doi: 10.3322/caac.21552, PMID: 30720861 PMC6403009

[58] Aerts HJ Velazquez ER Leijenaar RT Parmar C Grossmann P Carvalho S . Decoding tumour phenotype by noninvasive imaging using a quantitative radiomics approach. Nat Commun. (2014) 5:4006. doi: 10.1038/ncomms5006, PMID: 24892406 PMC4059926

[59] Lambin P Rios-Velazquez E Leijenaar R Carvalho S van Stiphout RG Granton P . Radiomics: extracting more information from medical images using advanced feature analysis. Eur J Cancer (Oxford England: 1990). (2012) 48:441–6. doi: 10.1016/j.ejca.2011.11.036, PMID: 22257792 PMC4533986

[60] Shin H Hur MH Song BG Park SY Kim GA Choi G . AI model using CT-based imaging biomarkers to predict hepatocellular carcinoma in patients with chronic hepatitis B. J Hepatol. (2025) 82:1080–8. doi: 10.1016/j.jhep.2024.12.029, PMID: 39710148

[61] Huang B Sollee J Luo YH Reddy A Zhong Z Wu J . Prediction of lung Malignancy progression and survival with machine learning based on pre-treatment FDG-PET/CT. EBioMedicine. (2022) 82:104127. doi: 10.1016/j.ebiom.2022.104127, PMID: 35810561 PMC9278031

[62] Xiang F Meng QT Deng JJ Wang J Liang XY Liu XY . A deep learning model based on contrast-enhanced computed tomography for differential diagnosis of gallbladder carcinoma. Hepatobil Pancreat Dis Int. (2024) 23:376–84. doi: 10.1016/j.hbpd.2023.04.001, PMID: 37080813

[63] Al Mohammad B Brennan PC Mello-Thoms C . A review of lung cancer screening and the role of computer-aided detection. Clin Radiol. (2017) 72:433–42. doi: 10.1016/j.crad.2017.01.002, PMID: 28185635

[64] Arun Kumar S Sasikala S . Review on deep learning-based CAD systems for breast cancer diagnosis. Technol Cancer Res Treat. (2023) 22:15330338231177977. doi: 10.1177/15330338231177977, PMID: 37282580 PMC10272643

[65] Leming MJ Bron EE Bruffaerts R Ou Y Iglesias JE Gollub RL . Challenges of implementing computer-aided diagnostic models for neuroimages in a clinical setting. NPJ Digital Med. (2023) 6:129. doi: 10.1038/s41746-023-00868-x, PMID: 37443276 PMC10345121

[66] Schwyzer M Martini K Benz DC Burger IA Ferraro DA Kudura K . Artificial intelligence for detecting small FDG-positive lung nodules in digital PET/CT: impact of image reconstructions on diagnostic performance. Eur Radiol. (2020) 30:2031–40. doi: 10.1007/s00330-019-06498-w, PMID: 31822970

[67] Chamberlin J Kocher MR Waltz J Snoddy M Stringer NFC Stephenson J . Automated detection of lung nodules and coronary artery calcium using artificial intelligence on low-dose CT scans for lung cancer screening: accuracy and prognostic value. BMC Med. (2021) 19:55. doi: 10.1186/s12916-021-01928-3, PMID: 33658025 PMC7931546

[68] Weikert T Akinci D’Antonoli T Bremerich J Stieltjes B Sommer G Sauter AW . Evaluation of an AI-powered lung nodule algorithm for detection and 3D segmentation of primary lung tumors. Contrast Media Mol Imaging. (2019) 2019:1545747. doi: 10.1155/2019/1545747, PMID: 31354393 PMC6636561

[69] Khan A Tariq I Khan H Khan SU He N Zhiyang L . Lung cancer nodules detection via an adaptive boosting algorithm based on self-normalized multiview convolutional neural network. J Oncol. (2022) 2022:5682451. doi: 10.1155/2022/5682451, PMID: 36199795 PMC9529389

[70] Choi W Oh JH Riyahi S Liu CJ Jiang F Chen W . Radiomics analysis of pulmonary nodules in low-dose CT for early detection of lung cancer. Med Phys. (2018) 45:1537–49. doi: 10.1002/mp.12820, PMID: 29457229 PMC5903960

[71] Hamm CA Wang CJ Savic LJ Ferrante M Schobert I Schlachter T . Deep learning for liver tumor diagnosis part I: development of a convolutional neural network classifier for multi-phasic MRI. Eur Radiol. (2019) 29:3338–47. doi: 10.1007/s00330-019-06205-9, PMID: 31016442 PMC7251621

[72] Kim DW Lee G Kim SY Ahn G Lee JG Lee SS . Deep learning-based algorithm to detect primary hepatic Malignancy in multiphase CT of patients at high risk for HCC. Eur Radiol. (2021) 31:7047–57. doi: 10.1007/s00330-021-07803-2, PMID: 33738600

[73] Yang Q Wei J Hao X Kong D Yu X Jiang T . Improving B-mode ultrasound diagnostic performance for focal liver lesions using deep learning: A multicentre study. EBioMedicine. (2020) 56:102777. doi: 10.1016/j.ebiom.2020.102777, PMID: 32485640 PMC7262550

[74] Mokrane FZ Lu L Vavasseur A Otal P Peron JM Luk L . Radiomics machine-learning signature for diagnosis of hepatocellular carcinoma in cirrhotic patients with indeterminate liver nodules. Eur Radiol. (2020) 30:558–70. doi: 10.1007/s00330-019-06347-w, PMID: 31444598

[75] Zhao X Liang P Yong L Jia Y Gao J . Radiomics study for differentiating focal hepatic lesions based on unenhanced CT images. Front Oncol. (2022) 12:650797. doi: 10.3389/fonc.2022.650797, PMID: 35574320 PMC9092943

[76] Ishioka J Matsuoka Y Uehara S Yasuda Y Kijima T Yoshida S . Computer-aided diagnosis of prostate cancer on magnetic resonance imaging using a convolutional neural network algorithm. BJU Int. (2018) 122:411–7. doi: 10.1111/bju.14397, PMID: 29772101

[77] Schelb P Kohl S Radtke JP Wiesenfarth M Kickingereder P Bickelhaupt S . Classification of Cancer at Prostate MRI: Deep Learning versus Clinical PI-RADS Assessment. Radiology. (2019) 293:607–17. doi: 10.1148/radiol.2019190938, PMID: 31592731

[78] Arif M Schoots IG Castillo Tovar J Bangma CH Krestin GP Roobol MJ . Clinically significant prostate cancer detection and segmentation in low-risk patients using a convolutional neural network on multi-parametric MRI. Eur Radiol. (2020) 30:6582–92. doi: 10.1007/s00330-020-07008-z, PMID: 32594208 PMC7599141

[79] Ahdoot M Wilbur AR Reese SE Lebastchi AH Mehralivand S Gomella PT . MRI-targeted, systematic, and combined biopsy for prostate cancer diagnosis. New Engl J Med. (2020) 382:917–28. doi: 10.1056/NEJMoa1910038, PMID: 32130814 PMC7323919

[80] Kim HE Kim HH Han BK Kim KH Han K Nam H . Changes in cancer detection and false-positive recall in mammography using artificial intelligence: a retrospective, multireader study. Lancet Digital Health. (2020) 2:e138–e48. doi: 10.1016/S2589-7500(20)30003-0, PMID: 33334578

[81] Rodríguez-Ruiz A Krupinski E Mordang JJ Schilling K Heywang-Köbrunner SH Sechopoulos I . Detection of breast cancer with mammography: effect of an artificial intelligence support system. Radiology. (2019) 290:305–14. doi: 10.1148/radiol.2018181371, PMID: 30457482

[82] van Winkel SL Rodríguez-Ruiz A Appelman L Gubern-Mérida A Karssemeijer N Teuwen J . Impact of artificial intelligence support on accuracy and reading time in breast tomosynthesis image interpretation: a multi-reader multi-case study. Eur Radiol. (2021) 31:8682–91. doi: 10.1007/s00330-021-07992-w, PMID: 33948701 PMC8523448

[83] Conant EF Toledano AY Periaswamy S Fotin SV Go J Boatsman JE . Improving accuracy and efficiency with concurrent use of artificial intelligence for digital breast tomosynthesis. Radiol Artif Intell. (2019) 1:e180096. doi: 10.1148/ryai.2019180096, PMID: 32076660 PMC6677281

[84] Pacilè S Lopez J Chone P Bertinotti T Grouin JM Fillard P . Improving breast cancer detection accuracy of mammography with the concurrent use of an artificial intelligence tool. Radiol Artif Intell. (2020) 2:e190208. doi: 10.1148/ryai.2020190208, PMID: 33937844 PMC8082372

[85] Xu H Tang RSY Lam TYT Zhao G Lau JYW Liu Y . Artificial intelligence-assisted colonoscopy for colorectal cancer screening: A multicenter randomized controlled trial. Clin Gastroenterol Hepatol. (2023) 21:337–46.e3. doi: 10.1016/j.cgh.2022.07.006, PMID: 35863686

[86] Glissen Brown JR Mansour NM Wang P Chuchuca MA Minchenberg SB Chandnani M . Deep learning computer-aided polyp detection reduces adenoma miss rate: A United States multi-center randomized tandem colonoscopy study (CADeT-CS trial). Clin Gastroenterol Hepatol. (2022) 20:1499–507.e4. doi: 10.1016/j.cgh.2021.09.009, PMID: 34530161

[87] Wang P Liu X Berzin TM Glissen Brown JR Liu P Zhou C . Effect of a deep-learning computer-aided detection system on adenoma detection during colonoscopy (CADe-DB trial): a double-blind randomised study. Lancet Gastroenterol Hepatol. (2020) 5:343–51. doi: 10.1016/S2468-1253(19)30411-X, PMID: 31981517

[88] Yamaguchi D Shimoda R Miyahara K Yukimoto T Sakata Y Takamori A . Impact of an artificial intelligence-aided endoscopic diagnosis system on improving endoscopy quality for trainees in colonoscopy: Prospective, randomized, multicenter study. Digest Endosc. (2024) 36:40–8. doi: 10.1111/den.14573, PMID: 37079002 PMC12136242

[89] Kamba S Tamai N Saitoh I Matsui H Horiuchi H Kobayashi M . Reducing adenoma miss rate of colonoscopy assisted by artificial intelligence: a multicenter randomized controlled trial. J Gastroenterol. (2021) 56:746–57. doi: 10.1007/s00535-021-01808-w, PMID: 34218329

[90] Mangas-Sanjuan C de-Castro L Cubiella J Díez-Redondo P Suárez A Pellisé M . Role of artificial intelligence in colonoscopy detection of advanced neoplasias: A randomized trial. Ann Intern Med. (2023) 176:1145–52. doi: 10.7326/M22-2619, PMID: 37639723

[91] Yuan XL Zhou Y Liu W Luo Q Zeng XH Yi Z . Artificial intelligence for diagnosing gastric lesions under white-light endoscopy. Surg Endosc. (2022) 36:9444–53. doi: 10.1007/s00464-022-09420-6, PMID: 35879572

[92] Nam JY Chung HJ Choi KS Lee H Kim TJ Soh H . Deep learning model for diagnosing gastric mucosal lesions using endoscopic images: development, validation, and method comparison. Gastrointest Endosc. (2022) 95:258–68.e10. doi: 10.1016/j.gie.2021.08.022, PMID: 34492271

[93] Xue P Tang C Li Q Li Y Shen Y Zhao Y . Development and validation of an artificial intelligence system for grading colposcopic impressions and guiding biopsies. BMC Med. (2020) 18:406. doi: 10.1186/s12916-020-01860-y, PMID: 33349257 PMC7754595

[94] Li Y Liu ZH Xue P Chen J Ma K Qian T . GRAND: A large-scale dataset and benchmark for cervical intraepithelial Neoplasia grading with fine-grained lesion description. Med Image Anal. (2021) 70:102006. doi: 10.1016/j.media.2021.102006, PMID: 33690025

[95] Kim S Lee H Lee S Song JY Lee JK Lee NW . Role of artificial intelligence interpretation of colposcopic images in cervical cancer screening. Healthcare (Basel Switzerland). (2022) 10. doi: 10.3390/healthcare10030468, PMID: 35326946 PMC8953422

[96] Yuan C Yao Y Cheng B Cheng Y Li Y Li Y . The application of deep learning based diagnostic system to cervical squamous intraepithelial lesions recognition in colposcopy images. Sci Rep. (2020) 10:11639. doi: 10.1038/s41598-020-68252-3, PMID: 32669565 PMC7363819

[97] Oura H Matsumura T Fujie M Ishikawa T Nagashima A Shiratori W . Development and evaluation of a double-check support system using artificial intelligence in endoscopic screening for gastric cancer. Gastric Cancer. (2022) 25:392–400. doi: 10.1007/s10120-021-01256-8, PMID: 34652556

[98] Kim BS Kim B Cho M Chung H Ryu JK Kim S . Enhanced multi-class pathology lesion detection in gastric neoplasms using deep learning-based approach and validation. Sci Rep. (2024) 14:11527. doi: 10.1038/s41598-024-62494-1, PMID: 38773274 PMC11109266

[99] Wu L He X Liu M Xie H An P Zhang J . Evaluation of the effects of an artificial intelligence system on endoscopy quality and preliminary testing of its performance in detecting early gastric cancer: a randomized controlled trial. Endoscopy. (2021) 53:1199–207. doi: 10.1055/a-1350-5583, PMID: 33429441

[100] Dong Z Wang J Li Y Deng Y Zhou W Zeng X . Explainable artificial intelligence incorporated with domain knowledge diagnosing early gastric neoplasms under white light endoscopy. NPJ Digital Med. (2023) 6:64. doi: 10.1038/s41746-023-00813-y, PMID: 37045949 PMC10097818

[101] Zech JR Badgeley MA Liu M Costa AB Titano JJ Oermann EK . Variable generalization performance of a deep learning model to detect pneumonia in chest radiographs: A cross-sectional study. PloS Med. (2018) 15:e1002683. doi: 10.1371/journal.pmed.1002683, PMID: 30399157 PMC6219764

[102] Subbaswamy A Saria S . From development to deployment: dataset shift, causality, and shift-stable models in health AI. Biostat (Oxford England). (2020) 21:345–52. doi: 10.1093/biostatistics/kxz041, PMID: 31742354

[103] Wang X Zhao J Marostica E Yuan W Jin J Zhang J . A pathology foundation model for cancer diagnosis and prognosis prediction. Nature. (2024) 634:970–8. doi: 10.1038/s41586-024-07894-z, PMID: 39232164 PMC12186853

[104] Park J Patterson J Acitores Cortina JM Gu T Hur C Tatonetti N . Enhancing EHR-based pancreatic cancer prediction with LLM-derived embeddings. NPJ Digital Med. (2025) 8:465. doi: 10.1038/s41746-025-01869-8, PMID: 40691317 PMC12280092

[105] Haver HL Ambinder EB Bahl M Oluyemi ET Jeudy J Yi PH . Appropriateness of breast cancer prevention and screening recommendations provided by chatGPT. Radiology. (2023) 307:e230424. doi: 10.1148/radiol.230424, PMID: 37014239

[106] Ghorbian M Ghobaei-Arani M Ghorbian S . Transforming breast cancer diagnosis and treatment with large language Models: A comprehensive survey. Methods. (2025) 239:85–110. doi: 10.1016/j.ymeth.2025.04.001, PMID: 40199412

[107] Bao H Yang S Chen X Dong G Mao Y Wu S . Early detection of multiple cancer types using multidimensional cell-free DNA fragmentomics. Nat Med. (2025) 31:2737–45. doi: 10.1038/s41591-025-03735-2, PMID: 40425843

[108] Cohen JD Li L Wang Y Thoburn C Afsari B Danilova L . Detection and localization of surgically resectable cancers with a multi-analyte blood test. Sci (New York NY). (2018) 359:926–30. doi: 10.1126/science.aar3247, PMID: 29348365 PMC6080308

[109] Zhou X Cheng Z Dong M Liu Q Yang W Liu M . Tumor fractions deciphered from circulating cell-free DNA methylation for cancer early diagnosis. Nat Commun. (2022) 13:7694. doi: 10.1038/s41467-022-35320-3, PMID: 36509772 PMC9744803

[110] Ahmed MA AbdelMoety A Soliman AMA . Predicting cancer risk using machine learning on lifestyle and genetic data. Sci Rep. (2025) 15:30458. doi: 10.1038/s41598-025-15656-8, PMID: 40830557 PMC12365227

[111] Chow R Drkulec H Im JHB Tsai J Nafees A Kumar S . The use of wearable devices in oncology patients: A systematic review. Oncologist. (2024) 29:e419–e30. doi: 10.1093/oncolo/oyad305, PMID: 37971410 PMC10994271

[112] Paiboonborirak C Abu-Rustum NR Wilailak S . Artificial intelligence in the diagnosis and management of gynecologic cancer. Int J Gynaecol Obstet. (2025) 171:199–209. doi: 10.1002/ijgo.70094, PMID: 40277295 PMC12353828

[113] Moadel AB Galeano D Bakalar J Garrett C Greenstone S Segev A . AI virtual patient navigation to promote re-engagement of U.S. inner city patients nonadherent with colonoscopy appointments: A quality improvement initiative. Front Oncol. (2024) 42:100. doi: 10.1200/JCO.2024.42.16_suppl.100

[114] Sylolypavan A Sleeman D Wu H Sim M . The impact of inconsistent human annotations on AI driven clinical decision making. NPJ Digital Med. (2023) 6:26. doi: 10.1038/s41746-023-00773-3, PMID: 36810915 PMC9944930

[115] Poniszewska-Marańda A Vynogradnyk E Marańda W . Medical data transformations in healthcare systems with the use of natural language processing algorithms. Appl Sci. (2023) 13:682. doi: 10.3390/app13020682

[116] Clark K Vendt B Smith K Freymann J Kirby J Koppel P . The Cancer Imaging Archive (TCIA): maintaining and operating a public information repository. J Digital Imaging. (2013) 26:1045–57. doi: 10.1007/s10278-013-9622-7, PMID: 23884657 PMC3824915

[117] Haider SA Borna S Gomez-Cabello CA Pressman SM Haider CR Forte AJ . The algorithmic divide: A systematic review on AI-driven racial disparities in healthcare. J Racial Ethnic Health Disparities. (2024) 18. doi: 10.1007/s40615-024-02237-0, PMID: 39695057

[118] Dankwa-Mullan I Weeraratne D . Artificial intelligence and machine learning technologies in cancer care: addressing disparities, bias, and data diversity. Cancer Discov. (2022) 12:1423–7. doi: 10.1158/2159-8290.CD-22-0373, PMID: 35652218 PMC9662931

[119] HIPAA privacy rule and public health . Guidance from CDC and the U.S. Department of health and human services. MMWR Suppl. (2003) 52:1–17, 9-20. 12741579

[120] Annas GJ . HIPAA regulations - a new era of medical-record privacy? N Engl J Med. (2003) 348:1486–90. doi: 10.1056/NEJMlim035027, PMID: 12686707

[121] Vlahou A Hallinan D Apweiler R Argiles A Beige J Benigni A . Data sharing under the general data protection regulation: time to harmonize law and research ethics? Hypertension. (2021) 77:1029–35. doi: 10.1161/HYPERTENSIONAHA.120.16340, PMID: 33583200 PMC7968961

[122] Han H . Challenges of reproducible AI in biomedical data science. BMC Med Genomics. (2025) 18:8. doi: 10.1186/s12920-024-02072-6, PMID: 39794788 PMC11724458

[123] Cutillo CM Sharma KR Foschini L Kundu S Mackintosh M Mandl KD . Machine intelligence in healthcare-perspectives on trustworthiness, explainability, usability, and transparency. NPJ Digital Med. (2020) 3:47. doi: 10.1038/s41746-020-0254-2, PMID: 32258429 PMC7099019

[124] Murdoch B . Privacy and artificial intelligence: challenges for protecting health information in a new era. BMC Med Ethics. (2021) 22:122. doi: 10.1186/s12910-021-00687-3, PMID: 34525993 PMC8442400

[125] Ziller A Mueller TT Stieger S Feiner LF Brandt J Braren R . Reconciling privacy and accuracy in AI for medical imaging. Nat Mach Intell. (2024) 6:764–74. doi: 10.1038/s42256-024-00858-y

[126] Nankya M Mugisa A Usman Y Upadhyay A Chataut R . Security and privacy in E-health systems: A review of AI and machine learning techniques. IEEE Access. (2024) 12:148796–816. doi: 10.1109/ACCESS.2024.3469215

[127] Bernstein MH Atalay MK Dibble EH Maxwell AWP Karam AR Agarwal S . Can incorrect artificial intelligence (AI) results impact radiologists, and if so, what can we do about it? A multi-reader pilot study of lung cancer detection with chest radiography. Eur Radiol. (2023) 33:8263–9. doi: 10.1007/s00330-023-09747-1, PMID: 37266657 PMC10235827

[128] Zeng A Houssami N Noguchi N Nickel B Marinovich ML . Frequency and characteristics of errors by artificial intelligence (AI) in reading screening mammography: a systematic review. Breast Cancer Res Treat. (2024) 207:1–13. doi: 10.1007/s10549-024-07353-3, PMID: 38853221 PMC11230971

[129] Zhu E Muneer A Zhang J Xia Y Li X Zhou C . Progress and challenges of artificial intelligence in lung cancer clinical translation. NPJ Precis Oncol. (2025) 9:210. doi: 10.1038/s41698-025-00986-7, PMID: 40595378 PMC12214742

[130] World Health Organization . Ethics and governance of artificial intelligence for health: WHO guidance (2024). Geneva: World Health Organization. Available online at: https://www.who.int/publications/i/item/9789240092375 (Accessed October 20, 2025).

[131] European Parliament and Council of the European Union . European Parliament and Council of the European Union. Regulation (EU) 2024/1689 of the European Parliament and of the Council of 13 June 2024 laying down harmonised rules on artificial intelligence (Artificial Intelligence Act). Off J Eur Union. (2024). Available online at: https://eur-lex.europa.eu/legal-content/EN/TXT/?uri=CELEX%3A32024R1689.

